# Technology and Social Media Use Among Patients Enrolled in Outpatient Addiction Treatment Programs: Cross-Sectional Survey Study

**DOI:** 10.2196/jmir.9172

**Published:** 2018-03-06

**Authors:** Robert D Ashford, Kevin Lynch, Brenda Curtis

**Affiliations:** ^1^ Addictions Department of Psychiatry University of Pennsylvania Philadelphia, PA United States

**Keywords:** digital divide, behavioral health, social media, addiction, recovery, relapse

## Abstract

**Background:**

Substance use disorder research and practice have not yet taken advantage of emerging changes in communication patterns. While internet and social media use is widespread in the general population, little is known about how these mediums are used in substance use disorder treatment.

**Objective:**

The aims of this paper were to provide data on patients' with substance use disorders mobile phone ownership rates, usage patterns on multiple digital platforms (social media, internet, computer, and mobile apps), and their interest in the use of these platforms to monitor personal recovery.

**Methods:**

We conducted a cross-sectional survey of patients in 4 intensive outpatient substance use disorder treatment facilities in Philadelphia, PA, USA. Logistic regressions were used to examine associations among variables.

**Results:**

Survey participants (N=259) were mostly male (72.9%, 188/259), African American (62.9%, 163/259), with annual incomes less than US $10,000 (62.5%, 161/259), and averaged 39 (SD 12.24) years of age. The vast majority of participants (93.8%, 243/259) owned a mobile phone and about 64.1% (166/259) owned a mobile phone with app capabilities, of which 85.1% (207/243) accessed the internet mainly through their mobile phone. There were no significant differences in age, gender, ethnicity, or socio-economic status by computer usage, internet usage, number of times participants changed their phone, type of mobile phone contract, or whether participants had unlimited calling plans. The sample was grouped into 3 age groups (Millennials, Generation Xers, and Baby Boomers). The rates of having a social media account differed across these 3 age groups with significant differences between Baby Boomers and both Generation Xers and Millennials (*P*<.001 in each case). Among participants with a social media account (73.6%, 190/259), most (76.1%, 144/190) reported using it daily and nearly all (98.2%, 186/190) used Facebook. Nearly half of participants (47.4%, 90/190) reported viewing content on social media that triggered substance cravings and an equal percentage reported being exposed to recovery information on social media. There was a significant difference in rates of reporting viewing recovery information on social media across the 3 age groups with Baby Boomers reporting higher rates than Millennials (*P*<.001). The majority of respondents (70.1%, 181/259) said they would prefer to use a relapse prevention app on their phone or receive SMS (short message service) relapse prevention text messages (72.3%, 186/259), and nearly half (49.1%, 127/259) expressed an interest in receiving support by allowing social media accounts to be monitored as a relapse prevention technique.

**Conclusions:**

To our knowledge, this is the first and largest study examining the online behavior and preferences regarding technology-based substance use disorder treatment interventions in a population of patients enrolled in community outpatient treatment programs. Patients were generally receptive to using relapse prevention apps and text messaging interventions and a substantial proportion supported social media surveillance tools. However, the design of technology-based interventions remains as many participants have monthly telephone plans which may limit continuity.

## Introduction

Mobile phone use has increased dramatically over the last decade. Today, 95% of US adults have a mobile phone, up from 66% in 2006. Mobile phone with app capability (smartphone) ownership has also more than doubled from 35% in 2011 to 77% in 2016 [1]. With the increase in mobile phone ownership, health care providers have become interested in integrating the use of mobile phones in the care of chronic conditions such as HIV, diabetes, hypertension, and asthma [2-6]. Mobile phones are portable, capable of receiving and transmitting data, and they are “always on.” They also offer health care providers the unique ability to connect with hard-to-reach populations that might otherwise not have access to health care services [7,8]. Mobile phones with app capabilities have the added benefit of being mini-computers that run software apps, connect to the internet, and have various embedded sensors. Sensors include Global Positioning Systems that monitor spatial location, accelerometers that record movement and gross motor activities, text that can be automatically analyzed using natural language processing to provide users with personalized feedback, and voice and tone records that can estimate mood [9]. Mobile phones also provide health care providers with a continuous stream of patient data regarding behavior, symptoms, and physiology. Recently, behavioral health apps have been developed that monitor psychiatric illnesses [10-14]. These apps have proven to be feasible across a wide range of conditions including schizophrenia [15], bipolar disorder [16], anxiety disorder [17], and depression [14,18,19].

Addiction researchers have recently begun to explore the use of mobile phones to support recovery from substance use disorders [20]. Alcohol and drug misuse was estimated to cost the nation over US $400 billion annually. In 2015, an estimated 7.7 million individuals in the United States had an illicit drug use disorder in the past year, but only 1.3 million individuals (approximately 17%) received substance use treatment [21]. Further, fewer than half of the patients who entered treatment completed it [22], with about 70% of patients experiencing a recurrence of use within a few months of initiating treatment [23].

Mobile phones can provide access to an assortment of online resources for attendees of outpatient substance use disorder treatment programs [24]. For example, patients can access the internet to search for local Alcoholics Anonymous (AA) and/or Narcotics Anonymous (NA) meetings, attend online AA/NA meetings, join an online recovery community, or download a recovery-based app to their mobile phone. Patients in treatment may also be able to stay connected to loved ones via phone and video calls, texting, and social networking sites, providing them with needed social support. These individuals can also use their phones to make medical appointments, communicate with health care providers, and communicate with potential employers.

Little is known about access to mobile phones and the use of digital platforms among patients attending outpatient substance use disorder treatment programs. McClure and colleagues examined the utilization of the internet and mobile phones with patients attending outpatient clinics, methadone/buprenorphine maintenance programs, and buprenorphine maintenance primary care clinics [25]. The majority of the patients reported access to a mobile phone (91%) and texting (79%). Patients also reported higher regular internet use (44%) than regular computer use (39%), which suggested that some patients were accessing the internet through their mobile phones, though information on the type of phone used was not collected in the study.

This paper explored the patterns of mobile phone usage, the use of Facebook, Twitter, and other social media platforms, and the receptiveness of using these types of digital platforms for interventions that promote positive recovery outcomes among patients attending community outpatient substance use disorder treatment programs. We also examined differences in digital platform use by age, gender, income, race, and preferred substances. The goals of these analyses were to assess disparities in digital media platform use and gain a better understanding of which platforms would be suitable for disseminating and sustaining real-world recovery promotion interventions for this population.

## Methods

### Recruitment

In May 2016, a self-administered, in-person, paper-and-pencil survey was conducted among patients attending outpatient substance use disorder treatment programs in Philadelphia. Participants were invited to participate by research staff between group sessions. These outpatient treatment programs treated approximately 800 patients monthly, all over 18 years of age. The requirements to participate in the survey were (1) current enrollment in the outpatient program at the time of the survey; (2) greater than 18 years of age; (3) no intellectual or developmental disability; (4) and willingness to provide informed consent to participate. The survey took 10 minutes to complete and no identifiable information was recorded to protect patient privacy. All study procedures were approved by the University of Pennsylvania Human Subjects Review Board.

### Survey

The survey included technology utilization questions, adapted from McClure and colleagues [25], to assess the communication patterns of patients enrolled in substance abuse treatment programs in the Baltimore area. The survey was updated to include questions pertaining to mobile phone ownership, social media usage, and interest in the use of digital platforms to monitor recovery (Multimedia Appendix 1).

Exposure level to drug cues and pro-recovery information on social media was measured via responses to the following items:

How often have you seen drug cues—things that made you want to use drugs on social media?

Responses ranging from 1 (always) to 5 (never)

How often have you seen recovery information on social media?

Responses ranging from 1 (always) to 5 (never)

Have you posted information on social media about being in recovery?

Yes/no

We measured receptiveness to the use of online platforms for interventions that promote positive substance use treatment outcomes via responses to the following items:

Do you think social media would be a good place to receive information to help you avoid relapse?

Yes/no

Would you join an online support group to help you during your recovery?

Yes/no

Would you join a Facebook support group to help you during your recovery?

Yes/no

Would you sign up to receive text messages to help you during your recovery?

Yes/no

Would you use an app placed on your phone to help your recovery from alcohol or substance use?

Yes/no

We also asked participants how they would like to access a digital outpatient treatment program to aide during recovery (website, social media, texting, app), and if they would you allow their social media accounts to be monitored to help prevent relapse.

### Data Analysis

The responses were entered into the data monitoring system using double entry. One research assistant entered the data while checking for mismatches and out-of-range values. A different research assistant then entered the same data again. The entries were compared via a computer that identified mismatches. When mismatches were identified, the data entry persons checked the original survey to determine the correct value(s).

All analyses were performed using SAS 9.3. The primary comparisons were of technology ownership and use responses over 3 age groups (Baby Boomers, Generation Xers, and Millennials), using binary and ordinal logistic regression models. These models included gender and race as covariates.

### Data Exclusion

Respondents were excluded from final analyses due to missing age, gender, race, and reporting zero use of technology. Respondents excluded due to missing demographic information totaled 6 (0.02%, 6/276) participants. Respondents excluded due to reporting zero use of technology totaled 11 (0.04%, 11/276) participants.

## Results

### Participants

Demographic information for study participants is shown in Table 1. The participants were 259 adults with a substance use disorder at the time of entrance to treatment at 4 Philadelphia area community-based intensive outpatient programs. The participants averaged 38.86 (SD 12.24) years of, primarily self-identified as male (72.9%, 188/259), African American (62.9%, 163/259), high school graduate or General Equivalency Diploma (GED; 58.8%, 152/259), single-never married (73.5%, 190/259), and unemployed (77.6%, 201/259) with a yearly income under US $10,000 (62.4%, 161/259). In addition, participants were stratified into the Millennial (18 to 35 years), Generation X (36 to 51 years), or Baby Boomer or older (52 or more years) generational categories that best mirror generational categories among the general population. Most participants were of the Millennial generation (46.3%, 120/259), followed by Generation X (32.4%, 84/259), and Baby Boomer or older (21.2%, 55/259). Participants cited marijuana (47.9%, 124/259) and alcohol (40.5%, 105/259) as preferred substances most frequently and the mean length of treatment was 4.64 (SD 8.35) months.

### Technology Ownership

Of the 259 participants, most owned a mobile phone (93.8%, 243/259), of which many were mobile phones with app capabilities (64.1%, 166/259), with no significant differences between generations (χ^2^_2_=1.39; *P*=.50). Among phone owners, mobile phone with app capability ownership differed significantly among generations (χ^2^_2_=17.62, *P*<.001); with Generation X (*P*=.001, OR 3.52 [95% CI 1.65-7.52]) and Millennial (*P*<. 001, OR 4.53 [95% CI 2.19-9.35]) generations being more likely to own a mobile phone with capabilities than Baby Boomers. No significant differences were found between Millennials and Generation Xers (*P*=.11, OR 0.78 [95% CI 0.40-1.51]). Among phone owners, provider plans differed significantly among generations (χ^2^_2_=10.25, *P*=.006); with Generation Xers (*P*=.02, OR 3.73 [95% CI 1.18-11.73]) and Millennials (*P*=.002, OR 6.09 [95% CI 1.94-19.09]) being more likely to have unlimited texting plans than Baby Boomers.

### Technology Use

The majority of all participants reported regularly using text messaging, email, the internet, and a computer. Generational differences were found to be significant in text message (χ^2^_2_=12.16, *P*=.002), email (χ^2^_2_=20.65, *P*<.001), and internet use (χ^2^_2_=26.37, *P*<.001), but not in computer use (χ^2^_2_=5.49, *P*=.06). The significant differences were largely due to the Baby Boomers using these media less than the Generation Xers or Millennials, with the Generation Xers tending to use less than the Millennials, but not significantly so (Multimedia Appendix 2).

Among those who accessed the internet, about 80.0% (152/190) reported that they typically accessed it via their mobile phone. There were no significant generational differences on accessing the internet by phone versus by some other means (χ^2^_2_=5.00, *P*=.08).

### Social Media Ownership and Use

Of the respondents, 73.6% (190/259) had a social media account of some type, with the majority using these accounts daily (76.1%, 144/190). There were significant generational differences (χ^2^_2_=38.25, *P*<.001); with both Generation Xers (*P*<.001, OR 5.62 [ 95% CI 2.62-12.03]) and Millennials (*P*<.001, OR 9.01 [95% CI 4.26-19.03]) being more likely to own a social media account. No differences were found between Millennials and Generation Xers (*P*=.19, OR 1.60 [95% CI 0.79-3.28]). There was a similar pattern of significant differences on frequency of use among participants with a social media account (χ^2^_2_=7.04, *P*=.03); with both Generation Xers (*P*=.02, OR 3.44 [95% CI 1.19-9.98]) and Millennials (*P*=.01, OR 3.81 [95% CI 1.38-10.59]) being more likely to have daily or weekly frequencies of use compared to Baby Boomers. No significant difference was found between Millennials and Generation Xers (*P*=.10, OR 1.11 [95% CI 0.51-2.41]).

**Table 1:** Demographic characteristics (N=259).

Characteristic		Value
Age in years, mean (SD)		38.86 (12.24)
**Generation, n (%)**		
	Millennials	120 (46.3)
	Generation X	84 (32.4)
	Baby Boomer	55 (21.2)
**Gender, n (%)**		
	Female	71 (27.0)
	Male	188 (73.0)
**Race, n (%)**		
	Nonblack	96 (37.1)
	Black	163 (62.9)
**Marital status, n (%)**		
	Single or never married	190 (73.5)
	Married or domestic partnership	29 (11.3)
	Widowed, divorced, or separated	40 (15.2)
**Education level, n (%)**		
	Did not complete high school	77 (29.6)
	High school graduate or GED^a^	152 (58.8)
	2-year degree or more	30 (11.7)
**Employment status, n (%)**		
	Employed	58 (22.4)
	Unemployed	201 (77.6)
**Income level per year, n (%)**		
	Less than $10,000	161 (62.5)
	$10,000 to $49,999	87 (33.8)
	Over $50,000	11 (3.8)
**Substance use^b^, n (%)**		
	Alcohol	105 (40.5)
	Opiates	73 (28.2)
	Cocaine	80 (30.9)
	Amphetamines	17 (6.6)
	Marijuana	124 (47.9)
Treatment length in months, mean (SD)		4.64 (8.35)

^a^GED: General Equivalency Diploma.

^b^Total is greater than 100% due to multiple responses from participants.

Virtually all participants with a social media account used Facebook (98.2%, 186/190), Instagram (60.22%, 114/190), Google+ (40.9%, 78/190), and Twitter (24.3%, 46/190) most frequently. Of these 4 main uses, there were significant generational differences for Twitter (χ^2^_2_=8.96, *P*=.03), Instagram (χ^2^_2_=24.68, *P*<.001), and SnapChat (χ^2^_2_=8.23, *P*=.02), but not for Google+ (χ^2^_2_=5.46, *P*=.07). Overall, Millennials made more use of all of the platforms than Baby Boomers or Generation Xers, with the exception of Google+.

Respondents predominantly used social media accounts to share photos and videos (83.4%, 158/190), stay in touch with family and friends (76.8%, 146/190), watch videos others post (70.2%, 133/190), instant message (67.4%, 128/190), and see updates about others (67.4%, 128/190).

### Substance Use and Recovery on Social Media

Among people with social media accounts, 47.4% (90/190) of respondents had seen information (eg, posts, text, images, videos, etc) that made them want to use substances at least sometimes on digital media platforms, with exactly the same percentage reporting that they had seen recovery information at least sometimes. A cross-tabulation showed no significant association between the people in each of the reporting groups (χ^2^_1_=3.20, *P*=.07), and this was true within generation groups (*P*>.11 in each generation). There was no significant difference among generations on seeing drug cues (χ^2^_2_=3.14, *P*=.21). However, there was a significant generational difference (χ^2^_2_=8.39, *P*=.02) on the frequency of seeing recovery cues. The Millennials were significantly less likely to see recovery information than the Baby Boomers (*P*=.01, OR 0.31 [95% CI 0.12-0.77]), with no significant difference between Generation Xers and Baby Boomers (*P*=.19, OR 0.5 [95% CI 0.20-1.36]), or between Millennials and Generation Xers (*P*=.07, OR 0.58 [95% CI 0.32-1.05]).

The majority of respondents (59.7%, 113/190) had not posted about their personal recovery on social media accounts, and this did not significantly differ among generations (χ^2^_2_=4.53, *P*=.11).

### Support on Social Media

Of the respondents, 66.1% (171/259) believed that social media platforms would be a good place to receive information to protect their recovery or prevent relapse. This belief did not differ across generations significantly (χ^2^_2_=6.11, *P*=.05). The majority of respondents (50.9%, 132/259) would not allow social media accounts to be monitored in order to support personal recovery, with no significant differences across generations (χ^2^_2_=2.34, *P*=.31).

Participants believed that providing support through social media (50.4%, 130/259) is preferred, compared to a website (36.8%, 95/259), text messaging (37.6%, 97/259), or mobile phone apps (37.2%, 96/259). However, a majority of participants would join an online support group (69.0%, 179/259), join a Facebook support group (62.3%, 161/259), sign up to receive text messages (71.9%, 186/259), or use an app placed on their mobile phone (70.4%, 182/259) to support their personal recovery. These differences were not significant among generations.

## Discussion

### Principal Findings

The results shown here demonstrate that technology adoption and internet use continues to rise, even among populations with substance use disorders. Previous research has shown that technological interventions using text message features [25] for substance using populations could be of benefit, though here we show that interventions delivered on social media platforms may be preferential. In addition, the “digital divide” that has been previously alluded to in the study of technological interventions in patients with substance use disorder [25], has been described to exist largely across racial lines. However, our results show that ethnic minorities present with rates similar to that of the general population in regards to mobile phone ownership and technology/internet use. The advent of mobile phones with capabilities has likely assisted in the partial bridging of this digital divide, also referenced as “digital differentiation” [26], supported by the prevalence of mobile phone ownership with app capabilities presented here (64.1%, 166/259).

The increased availability and use of social media platforms should also be viewed as potentially harmful to populations engaging in substance use disorder treatment. The majority of respondents in the current study had at least sometimes seen information that had resulted in the desire to return to substance use. Patients engaged in an outpatient setting that have regular access to social media and other digital platforms are at greater risks of encountering this information. The reported risk of relapse of patients in outpatient treatment settings has been as high as 70% [23]. With the risk of relapse for these populations already being high, the prevalence of those encountering triggering information on social media is high enough—47.4% (90/190) reported here—to support the increase in use of mediating supports, either in the form of recovery related information on similar platforms or targeted interventions using digital media.

Our results support that a recovery-focused social network may prove beneficial, especially to younger populations. Though this finding is age-specific, the Millennials and Generation Xers will soon make up the majority of people in substance use disorder treatment, suggesting that interventions and support services curtailed to this milieu is critical to positively improving treatment and recovery outcomes in the long-term.

Treatment centers offering substance use disorder specific or ancillary services should continue to inform themselves of potential benefits and harms of digital platforms as technology use and ownership continues to increase in all segments of the population, even those in lower socio-economic brackets. Factors such as these that have the potential to impact relapse vulnerability, outreach mechanisms, treatment engagement, and continuing aftercare should be discussed at length in service provision in the 21st century.

### Limitations

The sample consisted predominantly of black, low socio-economic status males. Though the sample is not reflective of the general population, the results are comparable with other technology use and ownership studies that have shown similar prevalence rates among other demographic cross-sections—while showing lower prevalence among minority communities. The results shown here suggest that technology adoption has increased exponentially among lower socio-economic status minority communities as it has become cheaper and more readily available. A secondary limitation is the geographical location of participants coming from one metropolitan area in the northeastern United States. Results are likely not generalizable to more rural areas of the United States, and the study should be replicated with a sample representative of these areas to confirm technology adoption and impacts on treatment and recovery from substance use disorders. In addition, the current study did not clearly delineate the types of self-defined information seen by respondents in regards to emotionally triggering or recovery-related information. Future studies should seek to identify the types of channels and format this information takes so that future targeted interventions can be better informed.

### Conclusions

Technology has continued to be adopted and used at increasing rates among all sectors of the population, including lower socio-economic status African-Americans. Seemingly ubiquitous mobile phone ownership and social media use among younger generations suggest that these platforms can have an immediate impact—potentially detrimental or beneficial—on an individual’s treatment and recovery from a substance use disorder. Substance use disorder treatment providers should consider the implications of technology ownership and digital media use in the modification of treatment protocols, where recovery-focused platforms can be used to impact relapse vulnerability, treatment engagement, and long-term recovery outcomes. Similarly, implementing provisions to mitigate the risk of drug-related cravings resulting from seeing drug-related information on digital media platforms should also be considered, especially for younger generation clients.

## Appendix

Multimedia Appendix 1 Survey instrument.

Multimedia Appendix 2 Odds ratio, generational differences in technology (**P*<.05).

## Abbreviations

AA: Alcoholics Anonymous
NA: Narcotics Anonymous
