# Supplementary figures and images for: Technology and Social Media Use Among Patients Enrolled in Outpatient Addiction Treatment Programs: Cross-Sectional Survey Study
Source: J Med Internet Res. 2018 Mar 6;20(3):e84. doi: 10.2196/jmir.9172 (PMC5861298; doi:10.2196/jmir.9172)

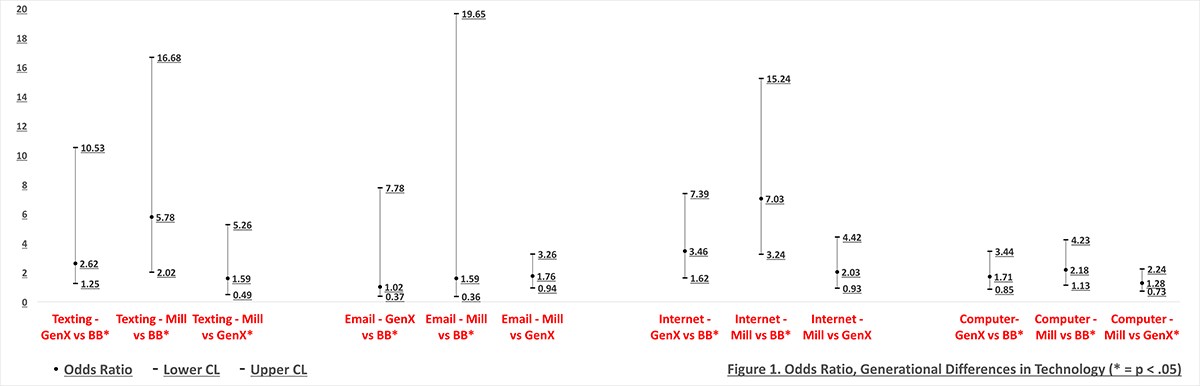

Supplement: Multimedia Appendix 2 [file jmir_v20i3e84_app2.png]
